# Primary care perspectives on implementation of clinical trial recruitment

**DOI:** 10.1017/cts.2019.435

**Published:** 2019-12-26

**Authors:** Teresa Taft, Charlene Weir, Heidi Kramer, Julio C. Facelli

**Affiliations:** 1 Department of Biomedical Informatics, University of Utah, Salt Lake City, UT, USA; 2 Center for Clinical and Translational Science, University of Utah, Salt Lake City, UT, USA

**Keywords:** Primary care, clinical trial recruitment, implementation, system design, FHIR

## Abstract

**Introduction:**

Poor clinical trial (CT) recruitment is a significant barrier to translating basic science discoveries into medical practice. Improving support for primary care provider (PCP) referral of patients to CTs may be an important part of the solution. However, implementing CT referral support in primary care is not only technically challenging, but also presents challenges at the person and organization levels.

**Methods:**

The objectives of this study were (1) to characterize provider and clinical supervisor attitudes and perceptions regarding CT research, recruitment, and referrals in primary care and (2) to identify perceived workflow strategies and facilitators relevant to designing a technology-supported primary care CT referral program. Focus groups were conducted with PCPs, directors, and supervisors.

**Results:**

Analysis indicated widespread support for the intrinsic scientific value of CTs, while at the same time deep concerns regarding protecting patient well-being, perceived loss of control when patients participate in trials, concern about the impact of point-of-care referrals on clinic workflow, the need for standard processes, and the need for CT information that enables referring providers to quickly confirm that the burdens are justified by the benefits at both patient and provider levels. PCP suggestions pertinent to implementing a CT referral decision support system are reported.

**Conclusion:**

The results from this work contribute to developing an implementation approach to support increased referral of patients to CTs.

## Introduction

Clinical trials (CTs) use the most rigorous standard scientific validation process to ascertain the effectiveness and safety of medical interventions [1]. However, problems with participant recruitment frequently lead to delays, or at times even result in the abandonment of approved trials [2, 3]. Insufficient recruitment has been found to be the leading cause of early termination of trials [4, 5]. The National Center for Advancing Translational Science (NCATS) has been charged with improving CT recruitment processes. In addition, a 2017 survey of Clinical and Translational Science Award Consortium members “Current Recruitment Practices” described the need for more research on the topic [6].

Recruitment through primary care providers (PCPs) may be part of the solution to insufficient recruitment as about 50% of patient visits occur in primary care settings [7]. PCPs frequently have an ongoing relationship with their patients and it is well documented that personal relationships and trust play a critical role in enrolling patients in CTs for rare diseases [8–10]. Expanding the role of PCPs to include more frequent referral of patients to CTs may significantly improve recruitment efficiency.

The US government’s passage of the 2009 HITECH Act has resulted in increased dissemination of electronic health records (EHRs) and has made possible more automated processes for identifying potential trial participants. These processes include alerts and tools to (1) prefilter eligible trial participants, (2) ascertain patient interest in study participation through patient portals, (3) request provider referral of specific patients, and (4) efficiently collect and share data [6, 11–13]. However, the use of computerized decision support for CT patient identification and recruitment is young. The technical design of these interventions may be somewhat straightforward; however, ensuring that primary care referral processes are embedded into the provider’s workflow is highly complex. These processes may affect many actors in the clinical care setting including patients, nurses, physicians, clinical coordinators, and investigators. Semi-automated recruiting for trials has reportedly been effective in some early studies [10, 14, 15]; however, implementation of these programs is highly variable making interpretation of the beneficial features difficult. Studies of effects of these Clinical Trial Recruitment Support Systems (CTRSS) frequently report on improved efficiency achieved by reducing man hours per trial participant or by increasing the number of participants referred. However, few report on physician factors, therefore psychological and behavioral factors are not fully understood [15].

Prior research studying PCP attitudes toward recruitment for CTs has found the following concerns: time constraints, lack of staff and training, worry about the impact on the doctor−patient relationship, concern for patients, loss of professional autonomy, difficulty with the consent procedure, lack of rewards and recognition, and a research question that is of little interest to the provider [16]. Implementing new, effective programs in primary care could produce significant benefits in improving CT recruitment. Of particular interest at this time of ubiquitous EHRs is whether provider concerns have changed and how emerging new technologies can be harnessed to mitigate concerns.

A 2012 systematic review reported some effective methods for increasing recruitment through primary care including peer-to-peer provider recruitment, enlisting colleague champions, assigning recruitment responsibility to researchers rather than providers, involving providers who share an interest in the research topic, averting duplication, simplifying patient eligibility criteria, using efficient methods to identify eligible patients (including database filtering), and reducing practitioner workload to afford greater research time [10]. However, patient referral to CTs is still low and the effectiveness of these recommendations integrated with CTRSS has not been fully assessed. This may be in part because of a lack of formal science-based metrics to evaluate recruitment effectiveness or because of a lack of comparative effectiveness research [6]. Effectiveness of CTRSS may depend most on how well the program is implemented, how the program is tailored to the local context, and the degree to which users find EHR tools to be useful and usable [14]. More research is needed in this area to optimize the use of CTRSS tools that will support provider workflow processes and will be adopted by PCPs.

## Objectives

Our overall goal was to inform implementation methods for CT recruitment programs in primary care. Our objectives were (1) to characterize provider and clinical supervisor attitudes and perceptions regarding CT research, recruitment, and referrals in primary care and (2) to identify perceived workflow strategies and facilitators relevant to designing a technology-supported CTRSS program in primary care. To accomplish these objectives, we conducted focus groups of PCPs and directors.

## Methods

### Focus Group Design

Focus groups have been shown to be effective in qualitative research and are particularly relevant when there is no right answer and the purpose of the investigation is to sample the range of perceptions. Focus groups support deeper discussion of issues and findings are especially helpful in designing new instruments. Factors associated with the validity of focus groups center around the degree to which diverse perspectives are elicited and valid group process is preserved. Sample size is not an issue in the traditional sense of minimizing beta error [17–19]. A script was created and reviewed for refinement by a nurse, a physician, three cognitive psychologists, and a biomedical informaticist, and then piloted with a group of four clinicians employed by the target organization. The final script used for each focus group discussion can be found in the supplementary material. The study was determined to be exempt by the Internal Review Board (IRB).

### Setting

The study was conducted in a tri-state, tertiary care, university-based healthcare system with 14 primary care clinics.

### Participants and Recruitment

Focus group participants were recruited from eight different primary care outpatient clinics through e-mail invitation. Topics were developed by four experienced investigators, with training in biomedical informatics, cognitive psychology, human factors, and workflow analysis, based on a knowledge of the literature. Participants were given an approved study cover letter and focus groups were conducted in meeting rooms during lunch breaks; a meal was provided. A total of six focus groups were conducted, with a range of 2–6 participants each. The 27 individual participants included 16 PCPs, 5 clinic directors, and 6 staff supervisors. Focus groups, which lasted approximately 30 minutes each, were conducted and audio-recorded by the research team.

### Qualitative Analysis

Focus group recordings were transcribed and an inductive thematic analysis was conducted using open coding to explore participant perceptions regarding their participation in patient recruitment for CTs. The authors (TT, CW, and HK) reviewed the transcripts, independently creating an initial set of pre-codes using the process recommended by Patton for inductive coding [20]. The set of pre-codes were iteratively discussed by the authors, regrouped, and organized into themes relevant to the design and implementation of a CT recruitment program. The potential themes were explored, independently and by consensus, over a series of three iterations until the final six themes were identified. An additional set of codes were extracted, which focused on the functional requirements for a CT referral system and included perceived information needs and experience with patient referral request methods. These codes were summarized through consensus.

## Results

The results are organized into two parts: thematic analysis and specific functional requirements identified by participants.

### Thematic Analysis Results

Six themes emerged from the qualitative analysis: (1) protection of patient well-being is paramount, (2) concern over loss of control of patient care decisions, (3) distrust in CT quality and relevance and the need for clinic-level oversight, (4) lack of time to locate and evaluate trial information, (5) need for standardized processes and consistency in clinical workflow, and (6) motivation increases with professional relevance, “What’s in it for me?” Examples of the participant statements that exemplify these discussion points are included below. Quotations have been minimally edited to correct spoken grammatical errors, protect anonymity, and improve readability [21].

#### Theme 1: Protection of Patient Well-Being is Paramount

Perceived benefits to the patient and protecting them from burdens and adverse health consequences seemed to be the providers’ greatest concern.

*If there’s something great that’s going to benefit a patient, I would definitely want to know about it to give them that option. That’s what we want to try to do, make our patients better*.

*If they don’t ever want to be contacted by a clinical trial– the patient experience really suffers if we’re constantly contacting them about a clinical trial*.

*With many patients, they don’t only have medical problems, but significant mental illness that sometimes interferes a lot with our treatment of them here for their clinical problems, and so, that probably would interfere with someone’s ability to understand and consent to a trial*.

*We don’t want to potentially put our patient at harm, if we already know there are things that can fix them …*.

#### Theme 2: Concern Over Loss of Control of Patient Care Decisions

The effect that patient participation in trials has on the provider’s ability to offer the best care for the patient was often a consideration.

*I’m not going to let you mess up my patient and then I’m going to have to deal with the consequences*.

*That’s the problem, we don’t know the effect. So, if we give a drug that might interact with whatever chemical they are testing, we’re not going to know. That could be an issue*.

*I don’t think our doctors would want them in a clinical trial because they are already seeing them, calling them, they come in for a care conference, the doctors get together with them, they talk about this patient; and if they said, “We are going to forget that, we are going to do a trial,” I think the doctors would say, “This is my patient and I’m controlling it*.”

*Someone who is really well controlled and doing well, I would not tend to put them toward the study, just keep doing what’s working right now*.

#### Theme 3: Distrust in Clinical Trial Quality and Relevance and the Need for Clinic-Level Oversight

Participants felt that CT screening at the clinic level, in addition to IRB oversight, was an important requisite in vetting and prioritizing CT studies they would support.

*If it’s just something to meet some quota for somebody that I don’t even know or care about, I don’t want to do it*.

*We have to also be very selective of the studies that come in because some of them aren’t written that well, and really don’t have much bearing on what we’re trying to do*.

*So, the IRB doesn’t understand what’s going on at [my clinic]*.

*I guess it would have to be local, local and reputable. I don’t want every little pharmaceutical company out there, trying to wheel and deal something, you know*.

*There probably should be a selection committee who can say, “Okay. Yeah, we’ll allow these studies to proceed forth [in our clinics].”*

#### Theme 4: Lack of Time to Locate and Evaluate Trial Information

Participants described difficulties in acquiring CT information in the time available during a patient visit and also a lack of funding allocated to support that work.

*My only concern would be if I’d start to be inundated with all these questions about these studies, “should I do it or not,” which I wouldn’t know about. I don’t have time to research it. “I really don’t, unless it’s something really, really significant. I mean, I have zero time.”*

*Our providers have 15 minutes with that patient and that is one of the biggest barriers, “I have 15 minutes and now this is taking ‘this many’ minutes away from my 15 minutes.”*

*I don’t think I would know enough. I think number one, it’s time consuming*.

#### Theme 5: Need for Standardized Processes and Consistency in Clinical Workflow

Participants were concerned with hardships that clinical staff face when asked to change their workflow.

*It can’t impact the visit, the efficiency and turn-around-time of the visit or it won’t work*.

*Sometimes it’s just a stability concern … if there’s no stability whatsoever, then people get frustrated and start to burn out*.

*It’s hard for us to remember [to refer patients] as we are flying through our day*.

#### Theme 6: Motivation Increases with Professional Relevance, “What’s In It for Me?”

Participants weighed the reciprocal benefits to their professional practice against the burden of assisting in CT recruitment.

*We’ve said no to some and yes to others, and it really has a lot to do with how relevant it is to what the main focus is, of the clinic*.

*There might be a whole slew of things that I never deal with or don’t care about… because it’s not prevalent for my patient population*.

*So just think what type of study, what the benefits are, if it’s hugely beneficial, because if it’s not, I don’t want it, because there are so many studies for everything*.

### Specific Suggestions

In this section, we report participant suggestions regarding a CTRSS program, organized into the following high-level categories: (1) referral information needs, (2) specific desired feedback, (3) workflow integration, and (4) referral notification methods.

#### CT Information Needs

Participants described information that would be important in helping them to make a referral decision (Table 1).

**Table 1.** Referral information needs

Trial Information
Study question and hypothesis
Inclusion and exclusion criteria
Study design (randomized, double-blind placebo, crossover …)
Benefits of participation
Risks of participation, potential side effects to watch for, and drugs to avoid
Intervention details including study drug class and protocol
Restrictions on care that will disqualify a participant
Study timeline including start and completion dates
Enrollment instructions
Study coordinator contact information
Link to patient information printout
Link to study website

#### Specific Desired Feedback

Participants felt it was reasonable to expect the feedback described in Table 2, when they had assisted with recruiting patients. Feedback served to decrease uncertainty and support patient care.

**Table 2.** Specific Desired Feedback

Feedback	Quote
Enrollment notification for patients who are participating in clinical trials, with information regarding trial interventions (especially for drug trials)	*I do like to know if they’re in [a trial] so that when they come in for problems, I at least know that they might be on a study medication, so I can be safe*. *I think it would be nice to have some type of alert saying, “The patient is enrolled in the trial,” and then I can click on it if I’m interested*.
Reports of effectiveness of provider recruitment efforts	*So, did we pick the right patient? … It’s helpful to know, “Did they even sign up for the study?”*
Clinical trial results	*A lot of times we do these and we just don’t hear anything about what transpired or what was fixed or what’s different…you never hear from them again… and I think that’s a demotivator*.
Professional recognition for involvement	*[I would like] recognition for the cutting edge of doing studies*

#### Workflow Integration

Participants described workflow supports, such as a clinic-level CT screening committee who review trials as part of their job (Table 3). Additional workflow supports including prescreening potential recruits prior to provider review, provision of CT information tailored to the recipient role, and an efficient referral decision response process were desired. Many of these involve interventions at the clinic administration level.

**Table 3.** Workflow integration suggestions

Suggestion	Quote
Site-level screening and vetting process to limit the number of trials that individual providers deal with	*Having a research committee… where you have representatives from the clinics, operations manager, medical director, or providers that would get together monthly and say, “Well, how much are we doing and what– how much of these requests are we going to be able to fill and where could they be?”*
Training of clinic staff, tailored to their role in supporting trials	*I think you have to have varying levels of education. Your physicians are going to have to know way more than your front-end staff do, and so your education piece has to be tailored to their involvement.*
Minimizing impact on clinic staff workload	*It has to be something really simple that someone else can help us with that we can just say yes or no to*.
Electronic links to useful information including patient printouts	*There should be some link to print off handouts to give to the patient so you don’t have to spend a lot of time explaining*.
Handoff must be efficient and straightforward	*Having somebody else do the explaining, the biggest part of the recruiting, and arranging so that all we have to do is identify the patient and hand them off*
Prescreening of recruits to narrow provider review	*Someone specifically doing a research study screened through all my patient’s and found like 1 or 2. That was fine for me*.

#### Referral Notification Methods

Participants suggested methods and shared perspectives on being contacted for CT patient referral requests (Table 4). Responses are grouped into positive and negative sentiment.

**Table 4.** Suggested referral request notification methods

Notification method	Positive comments	Negative comments
E-mail	*Email is another good way, especially if it’s a list of patients, and you can just quickly check, check, check, check; send it back*.	*E-mails are awful. We just get so far behind*.
Electronic health record In-basket message	*I would prefer it in the in-box cause it’s easier to link that [to the patient record] right away*.	*We have so many more folders in our in-baskets of refills and labs and phone calls and ‘close your chart’s and it [a clinical trial patient referral request] would be last. It just wouldn’t get done*.
Staff meeting	*Every once in a while, somebody could come and talk at the provider meeting to reach all the providers at once*.	*If the provider meeting fills up with 4 or 5 people that want to come and talk about studies, we don’t have time to talk about the things that we’re doing with our quality projects, things that are happening at the clinic level*.
Text message	*They used to text me when the patient was a possible recruit, so I used to text them back, “Yes.” That’s as simple as that*.	*None mentioned*.
Electronic medical record alert	*In an ideal world it might be nice to have a pop-up, “This patient might be ideal for this study. Is it okay if we contact them? Yes or No?” It helps us to be able to identify the patients, but not have to spend extra time in our already overburdened visit trying to talk about something that we don’t have all the details on*. [Best time to see alert] *When you’re reviewing the chart before you go into the room*. *As soon as you open the patient’s chart*	*If you have a training and some people are absent…they aren’t going to understand how to handle it*. *We’ll be honest that everybody gets alert fatigue and they start ignoring them*. *Hopefully they’re not getting alerts for 20 different types of studies*. *There may be physicians that want to opt in or out of pop-ups.* *Can it be set up so when a patient says, "No," or whatever happens, that alert doesn’t keep coming, so we don’t ask multiple times*.

### Summary

In summary, participants indicated widespread belief in the intrinsic scientific value of CTs, but at the same time revealed deep concern regarding protecting patient well-being. Loss of control when patients participate in trials, the impact of point-of-care referrals on clinic workflow, the need for standard processes and the need for information to allow referring providers to confirm that the burdens are justified by the benefits both at the participant and provider level were also identified. Analysis suggests that improvement to CT recruitment implementation in primary care is needed at the person, organization, and technical levels.

## Discussion

We identified important insights into PCP and clinic supervisor attitudes and perceptions regarding CT recruitment in primary care. In addition, we identified perceived workflow barriers and facilitators relevant to designing a program of research recruitment that includes EHR tools. Of great interest may be PCP perspectives on CT referral requests and the data that should be presented, strategies for clinic-wide implementation, and provider views on what makes a CT valuable. The attitudes identified are similar to those from Ross’s 1999 study [16], so it appears that the addition of the EHR into clinical practice has not dramatically changed the endemic problems faced by PCPs in referring patients to CTs. The participants’ suggestions addressed many of these concerns. We propose that IT-based approaches could be used to address and/or mitigate many of these more complex issues.

### Person Level: Primary Care Culture and Practice

PCPs participating in this formative evaluation described their commitment to protecting their patients’ well-being. Because PCPs adhere to a value system of delivering ongoing, patient-centered, comprehensive care, they often feel responsible for counseling patients and coordinating all aspects of their care [22]. This broad-level sense of responsibility may be unique to primary care. It is likely, therefore, that any program for primary care CT recruitment will have to take this deep-seated cultural aspect of primary care professional attitudes into account. There was a concern over burdening patients with repeated invitations to participate in trials when they have indicated that they are not interested. As described by Weng *et al*. [23], automated tracking of patient readiness and willingness to be recruited may be an important component of reassuring PCPs that they are not burdening their patients with these requests. In this study, providers were concerned that patient CT participation had, at times, reduced their ability to effectively manage the care of patients for whom they have assumed responsibility. Endorsing participation in a randomized trial may, therefore, feel unethical when a patient is doing well under current treatment or when management of the patient’s problems seems straightforward [24].

Significant uncertainty may exist for PCPs as they try to understand the validity of a specific CT treatment, protocols (including randomization), patient burdens, and other trial details. This uncertainty may create a tension with the PCP’s need for control and autonomy in their practice. However, it may be possible to enhance control by providing efficiently digestible, standardized CT information about CTs.

Results from this study indicate that PCPs need CT information at three times: (1) prior to or during the patient visit for referral decision-making, (2) when a patient is enrolled in a trial, and (3) after the trial has been completed. To reduce uncertainty at the time of the referral decision, CT information should be provided that is concise, yet detailed enough for the physician to quickly confirm that the benefits of participating justify the risks. To reduce PCP uncertainty when patients are enrolled in a CT, there should be a clear indicator in the patient’s medical record indicating trial participation and providers should have quick access to the study timeline, the trial interventions, risks to watch for, and CT investigator contact information. To reduce uncertainty about what happened as a result of patient referral, timely reciprocal feedback on effectiveness of recruitment efforts, trial progress, and trial results should be given. This information could be efficiently delivered via well-established EHR means, like Infobuttons, that can provide just in time contextual information [25, 26]. Provision of these items may go a long way toward reducing the uncertainty that is created when, “*We do these and we just don’t hear anything about what transpired or what was fixed or what’s different…you never hear from them again*.”

### Organizational Level: Workflow Integration Support

Difficulties such as time constraints and concern over managing variable workflow processes were described by our focus group participants and may contribute to a feeling of loss of control. These findings are supported by numerous other studies [16]. Because the median time allotted for a primary care visit is just 14.9 minutes [27], standardized processes are needed to support more efficient patient referral to CTs. Many recruitment efforts may fail because they do not consider system-level workflow issues in the implementation process.

CT investigators may provide clinic-level support by (1) providing funding for site review and vetting of their study, (2) fostering efficient handoffs of participants, (3) providing timely feedback regarding recruitment efforts and trial progression and outcomes, and (4) inputting trial data into a site’s CT template to support efficient, standardized viewing of trial information, and automated pre-screening. Online registries of trial information are becoming available which may be useful. For example, many CTs are now registered at clinicaltrials.gov and Infobutton links to this data could be included in the EHR. However, readability of the trial information on clinicaltrials.gov is often very difficult [28]. Human factors and usability evaluations are needed to improve the usefulness of clinicaltrials.gov and locally implemented CT referral displays. Using formal process modeling techniques may allow for the reengineering of clinical processes to deliver clinical care that is still efficient but is also more resilient to CT recruitment interventions.

Introducing CT referral interventions in the primary care setting requires attention to supporting the provider’s ability to maintain control of the patient’s healthcare. There is a need for improved communication between CT investigators and providers, which includes the efficient provision of specific, focused trial information prior to recruitment, and informing PCPs of any problems or concerns that arise after a patient has been recruited to a CT. In addition, referring PCPs should receive results once the CT is completed [22, 29]. Health information technology holds many of the keys to supporting efficient information exchange that can help to foster this communication; however a multifaceted approach to increasing PCP referral of patients to CT is likely required [6, 30].

### Technical Level: Design of Referral Requests

Information technology designed to reduce clinician burdens may support PCP referral of patients to CTs. Standardizing the display and process is important for reducing cognitive demands and for streamlining the process to preserve time allocated to other responsibilities. Automated prescreening, integration support for standard CT referral displays and notification request methods are described below.

#### Automated Prescreening

Computer algorithms that make use of artificial intelligence to analyze patient records can be employed to limit the number of potential recruits that a provider is asked to review for CT recruitment and to improve efficiency of those efforts [31–33]. Use of these prescreening methods is a recommended first step in any primary care semi-automated recruitment effort [6, 11–13] prior to requesting screening by providers. The integration of vendor agnostic CT referral applications for use in limited EHRs may be needed.

#### Standard Display

Providers may receive referral requests from multiple CT coordinators who use different third-party electronic data systems. Study participants indicated a need for consistency in their workflow, which includes the way they interact with referral requests and view CT data. Integrative technology is necessary to support PCPs use of a single CT referral display to view CT data, regardless of which third-party application was used to create it. One promising solution to this integration challenge is SMART on FHIR (Substitutable Medical Applications and Reusable Technologies on Fast Healthcare Interoperability Resources), which combines the SMART standards-based technology platform with HL7’s FHIR data interoperability standard [34]. This government-funded open platform supports the competitive development of substitutable, vendor independent third-party applications. Major EHR vendors are actively supporting the use of integrative technologies and the integration of third-party apps with their systems [35–38].

Standardization of the CT display can minimize burdens by improving not only the efficient location of information, but also the internalization of behavioral scripts that drive action and are prompted by critical environmental cues [39–40]. These scripts, “When this happens, then I do this,” are essential for managing the fast-paced context of primary care visits, where there is often little time or stamina for nonessential laborious mental processing.

#### Referral Request Methods

A variety of notification methods have been used for CT recruitment programs. Notifications may be pulled by providers at a time they choose or they may be configured to be interruptive reminders. Pop-up alerts embedded in the EHR are an interruptive notification method that have been widely used for decision support and have been alternately heralded as a breakthrough and criticized as a cause of physician burnout [41–43]. Initial introduction of pop-up EHR alerts requesting referral of pre-screened patients to CTs often produce increased PCP referral rates when they occur in a specialty clinic where other alerts rarely occur [23, 44, 45]. However, these results may be greatly reduced in a clinical context of multiple alerts. Alerts may only be helpful when they are prepared for, expected, understood, and when they are viewed as useful in completing important tasks [46].

Alternative methods to pop-up CT alert notification and referral requests include face-to-face personal or staff meetings; e-mail, in-basket or text messages; an EHR CT dashboard; and postal mail notification. The number of referral requests and the design of the referral notification and response system must respect provider time and provide needed information as efficiently as possible within the needs of the local health system. To accomplish this, each clinical site should have in place a semi-automated trial prescreening process that evaluates trials for factors that justify involvement of their providers and staff. Health systems should adopt a uniform CT referral request notification display, which has been developed and tested using iterative human factors design practices.

### Strengths and Limitations of this Approach

Focus group methods are frequently used for early identification of functional requirements and program implementation as they capture shared perspectives and have been shown to be effective for identifying perceptions, attitudes, information needs, and other human factors effecting workflow [19, 17]. However, there is a risk that more vocal participants will dominate, suppressing alternative points of view. To accommodate for this risk, focus groups were kept small and six separate focus groups were conducted.

Generalizability of the findings may be limited because participants were not randomly selected, but were recruited based on their willingness to volunteer and all focus groups were piloted and conducted within primary care clinics of one healthcare organization. Gender and race data were not collected, so we cannot comment on the diversity of the panel. However, attitudes identified in our focus groups align well with prior studies that have reported barriers to provider assisted recruitment including: time constraints; lack of staff and training; worry about the impact on the doctor−patient relationship; concern for patients, loss of professional autonomy; difficulty with the consent procedures; lack of rewards and recognition; and an insufficiently interesting research question, the need to clearly communicate to providers the rationale for the research, and potential patient and professional benefits [16, 47].

### Conclusion

Improving CT recruitment in primary care involves designing a complex, multifactorial program that addresses provider attitudes, workflow demands, and attention to IT design. Implementation requires attention to the whole process of CT referral at the person, organization, and technical level including notification, decision support, feedback, and CT information access. Clinic-level strategies must be integrated with the larger institution rules for providing resources and support.

## Supplementary material

For supplementary material accompanying this paper visit http://dx.doi.org/10.1017/cts.2019.435. click here to view supplementary material
