# Supplementary material for: Primary care perspectives on implementation of clinical trial recruitment
Source: J Clin Transl Sci. 2019 Dec 26;4(1):61–8. doi: 10.1017/cts.2019.435 (PMC7103461; doi:10.1017/cts.2019.435)
Supplement: Supplementary file 1 [file S2059866119004357sup001.docx]

Focus Group Questions

1. Have you ever been asked to help recruit patients to a clinical study? What was your response and why?
2. Have you ever had a conversation with a patient about their participation in a randomized clinical trial? Would you be willing to describe for us how that went and what you did?
3. What factors do you consider before notifying your patient that they may qualify for a study?
4. What are the benefits and barriers to informing patients at your clinic that they may qualify to participate in a study?
5. How would you feel about receiving an alert, during a visit, informing you that your patient may qualify for a clinical study? When is the best time to see that alert?
6. What information would you need to see before asking your patient for permission to be contacted by a study recruiter?
7. Who, in your clinic, would be best able to get a patient’s permission to be contacted regarding trial recruitment?
8. If your patient agreed in [the patient portal] to be contacted about participating in a trial, would you want to be notified prior to that happening?

*Due to time limitations, all questions may not be fully discussed.
